# Neurovascular Coupling Dysfunction: An Early Indicator of Brain Injury in Asymptomatic Moyamoya Disease

**DOI:** 10.2174/011570159X440563260226104506

**Published:** 2026-05-04

**Authors:** Rui Hu, Jinhao Lyu, Ziqi Liu, Qi Duan, Chaobang Xie, Xiaokuan Hao, Caohui Duan, Song Wang, Xiangbing Bian, Jianxing Hu, Rong Wang, Lian Duan, Xin Lou

**Affiliations:** 1 Department of Radiology, The First Medical Center of Chinese PLA General Hospital, Beijing, China;; 2 Department of Neurosurgery, Beijing Tiantan Hospital, Capital Medical University, Beijing, China;; 3 Department of Neurosurgery, The Fifth Medical Center of Chinese PLA General Hospital, Beijing, China

**Keywords:** Asymptomatic moyamoya disease, neurovascular coupling, diffusion tensor imaging, normal signals, cognitive, bilateral, unilateral

## Abstract

**Introduction:**

This study investigated structural and functional impairments in asymptomatic Moyamoya Disease (MMD) patients with normal signals to support early interventions. It focused on Neurovascular Coupling (NVC), cerebral perfusion, neural activity, and white matter microstructure in adults with unilateral and bilateral MMD, examining their impact on cognitive function.

**Methods:**

119 asymptomatic MMD adults (57 unilateral, 62 bilateral) underwent MRI assessments. Data on demographics, clinical, and neuropsychological parameters were collected. Comparative analyses of Cerebral Blood Flow (CBF), white matter diffusion, neuronal activity, and NVC were conducted, with Spearman correlation analyses assessing the link between imaging differences and cognitive function, adjusting for multiple comparisons.

**Results:**

The study identified that patients with bilateral asymptomatic MMD had lower NVC across two coupling modes (*P* = 0.004/0.049) and fractional anisotropy within two clusters (*P* = 0.028/0.039), alongside higher radial diffusivity in three clusters (*P* = 0.046) compared to those with unilateral involvement. While neuronal activity differed, CBF changes were not significant. NVC demonstrated a positive correlation with fractional anisotropy (*P* = 0.005) and was negatively related to radial diffusivity across two clusters (*P* = 0.025/0.018). Furthermore, both fractional anisotropy (*P* = 0.031) and NVC were positively associated with cognitive performance (*P* = 0.003).

**Discussion:**

This study shows that in asymptomatic MMD patients with normal signals, bilateral vascular involvement leads to more severe NVC impairments, despite similar CBF levels. This condition may worsen white matter damage and increase the risk of cognitive decline and adverse outcomes as the disease progresses. Early detection of NVC dysfunction could indicate a higher risk of future clinical issues and guide decisions on vascular reconstruction surgery. These findings suggest that NVC is an earlier indicator of brain dysfunction than traditional CBF measures in asymptomatic MMD patients.

**Conclusion:**

Bilateral asymptomatic MMD is associated with greater NVC impairment, possibly worsening microstructural damage and cognitive decline, suggesting a basis for targeted interventions.

## 1 INTRODUCTION

Moyamoya Disease (MMD) is a distinctive cerebrovascular disorder characterized by the progressive stenosis of the distal segments of the internal carotid arteries and the formation of atypical collateral vessels [1-3]. At the time of diagnosis, some individuals may present as asymptomatic, despite imaging examinations revealing significant vascular lesions in the absence of overt neurological symptoms or signs [4-7]. Nevertheless, these asymptomatic individuals remain at risk for future stroke or cognitive decline.

Numerous studies have demonstrated alterations in the microstructure and function of the brain in patients with MMD [8-12], which may correlate with cognitive impairment [13-18], and such alterations could potentially be present even during the asymptomatic phase [19-22]. Research on asymptomatic MMD patients has identified cognitive deficits associated with factors such as white matter hyperintensities [23-26]. It is important to note that prior studies have examined symptomatic or signal-abnormal asymptomatic MMD.

However, it remains unclear whether there is microstructural and functional damage in asymptomatic moyamoya disease patients with normal brain signal expression; therefore, this study focuses on asymptomatic patients with normal signals. Such microscopic injuries are not easily detected in routine imaging examinations. Advanced imaging technologies and analysis methods, including Diffusion Tensor Imaging (DTI) and Neurovascular Coupling (NVC) can provide a better evaluation of the brain health status and reserve capacity of these patients [9, 27, 28]. Early identification and monitoring of these potential changes are of great significance for asymptomatic MMD intervention and management [29, 30].

In the clinical management of asymptomatic MMD, there is an increasing interest in the focus on unilateral and bilateral vasculopathy, despite the limited availability of related clinical evidence [31, 32]. It is hypothesized that patients with unilateral vascular involvement may compensate through the relatively normal vasculature on the contralateral side, whereas those with bilateral involvement may experience significantly reduced compensatory capacity. Nonetheless, the distinct micro-level brain health characteristics of asymptomatic MMD patients with unilateral versus bilateral vascular involvement remain inadequately understood.

Consequently, this study aims to investigate differences in NVC, Cerebral Blood Flow (CBF), spontaneous neural activity, and white matter microstructure between unilateral and bilateral vascular involvement in asymptomatic MMD in adults with normal-appearing brain signals. Additionally, the study explores the relationship between these imaging indicators and cognitive function.

## 2 MATERIALS AND METHODS

### 2.1 Participants and Clinical Assessment

In accordance with the Declaration of Helsinki, the Ethics Committee of the Chinese PLA General Hospital has thoroughly reviewed and approved our research, granting it the approval number S2023-223-01.

Participants for this prospective cohort study were recruited from two centers located in Beijing. Eligible Patients were those diagnosed with asymptomatic MMD *via* cerebral angiography and MRA, exhibiting either bilateral or unilateral vascular involvement, and without a history of transient ischemic attacks, ischemic strokes, hemorrhagic strokes, or involuntary movements related to the disease. Additionally, the brain parenchyma of these patients appeared normal, as evidenced by the absence of abnormally elevated signals on standard T2-weighted imaging. Patients displaying abnormal parenchymal signals were excluded from the study. Moreover, individuals diagnosed with quasi-MMD associated with underlying conditions, as well as those unable to undergo MRI examinations, were also excluded.

Between January 2023 and April 2025, the Chinese PLA General Hospital undertook a prospective recruitment of patients diagnosed with MMD. The final analysis cohort comprised 119 cases of adult asymptomatic MMD, each with clinically confirmed normal brain signals. The criteria for subject inclusion and exclusion are detailed in Fig. (**1**). Clinical data, including age, gender, years of education, vascular risk factors, and cognitive assessments, were collected from the participants. However, only 61 individuals completed a comprehensive cognitive assessment. This assessment primarily encompassed the Cognitive Reflection Test (CRT), the Verbal Working Memory-forward digit span task (VWM1), the Verbal Working Memory-backward digit span task (VWM2), and the visual search task.

### 2.2 Image Acquisition

All participants underwent conventional MR sequences and the high-resolution three-dimensional T1-weighted imaging (3D-T1), 3D pseudocontinuous arterial spin labeling (3D pCASL), DTI, and resting-state functional MRI (rs-fMRI) utilizing a 3.0-Tesla MRI scanner. DTI data acquisition involved diffusion encoding in 35 directions with a single b-value (b = 1000 s/mm^2^) and one non-diffusion-weighted image (b = 0 s/mm^2^), with the following parameters: Repetition Time (TR) = 6000 ms, Echo Time (TE) = 62.9 ms, Field of View (FOV) = 240 mm × 240 mm, matrix size = 128 × 128, number of excitations = 1, slice thickness = 3 mm, and no inter-slice gap. The 3D pCASL data were obtained with the parameters: TR = 4844 ms, TE = 10.5 ms, FOV = 240 mm × 240 mm, slice thickness = 4.0 mm, no slice gap, and a post-labeling delay of 2.0 s. For rs-fMRI data acquisition, the parameters were: flip angle = 90^o^, TR = 2000 ms, TE = 30 ms, FOV = 240 mm × 240 mm, matrix size = 80 × 80, slice thickness = 3 mm, and no slice gap. The 3D-T1 data were collected with the following parameters: flip angle = 15^o^, TE= 3.0 ms, TR= 7.0 ms, FOV = 240 mm × 240 mm, matrix size = 256 × 256, and a slice thickness of 1.0 mm with no gap.

### 2.3 DTI Preprocessing and Tract-based Spatial Statistics Analysis

In the preprocessing stage of imaging analysis, a standardized procedure was consistently followed, and strict quality control measures were implemented throughout the entire process to ensure reproducibility.

The Brain Software Library for Functional MRI (FSL) was used for the processing of DTI data [33]. This process included the correction of eddy current distortions, the management of motion artifacts, and the execution of skull stripping. FSL was further utilized to compute DTI parameters, such as Fractional Anisotropy (FA), Radial Diffusivity (RD), Axial Diffusivity (AD), and Mean Diffusivity (MD). For voxel-wise statistical analysis of FA maps, the Tract-Based Spatial Statistics (TBSS) toolbox within the FSL suite was employed. FA images from all participants were spatially normalized to the Montreal Neurological Institute (MNI) space [34]. A mean FA image was subsequently generated, from which a white matter tract skeleton was derived. During the skeletonization process, an FA threshold of 0.20 was implemented to minimize the influence of gray matter and cerebrospinal fluid and to reduce intersubject variability [35, 36]. Aligned FA images from all participants were then projected onto this skeleton. The transformations applied to FA images were similarly applied to MD, AD, and RD images for subsequent statistical analyses. A voxel-wise analysis was conducted utilizing the “Randomize” command in FSL to assess differences among the four parametric images between the two groups within a General Linear Model (GLM), incorporating adjustments for covariates such as age, gender, and years of education. A total of 5000 permutations were executed using the mean FA skeleton as a mask [37]. The Threshold-Free Cluster Enhancement (TFCE) option in FSL was applied to determine the significance threshold, which was set at *P* < 0.05 following correction for family-wise error.

### 2.4 3D pCASL Processing

For the processing of CBF maps obtained from 3D-pCASL, the SPM12 software package was employed [38, 39]. Initially, the CBF maps were aligned with the corresponding 3D-T1 images. Following registration, segmentation of cerebrospinal fluid, white matter, and gray matter was performed on the 3D-T1 images, generating a deformation field for each participant. Subsequently, the CBF images for each participant were spatially normalized to the standard MNI template space using the corresponding deformation fields [40, 41]. Data normalization was carried out using the DPABI software to ensure dataset consistency, and the SPM software was utilized to smooth the normalized CBF images.

### 2.5 rs-fMRI, and NVC Processing

The RESTplus software package was used to compute four indicators of rs-fMRI: Amplitude of Low Frequency Fluctuation (ALFF), fractional ALFF (fALFF), Regional Homogeneity (ReHo), and Degree Centrality (DC) [42, 43]. The preprocessing steps encompassed the conversion of DICOM to NIFTI format, exclusion of the initial 10 time points (yielding 170 time points), slice timing correction, realignment, reorientation, normalization (involving co-registration of the fMRI images with corresponding T1-weighted anatomical images), smoothing, detrending, regression of nuisance covariates, and temporal filtering [44, 45]. A motion correction threshold was established at 3°. Following this, postprocessing is performed to calculate the ALFF, fALFF, ReHo, and DC.

 At the global gray matter level, four neurovascular coupling modes (CBF-ALFF, CBF-fALFF, CBF-ReHo, CBF-DC) were identified using the DPABI software package. (http://rfmri.org/dpabi). To calculate NVC, it is imperative to have previously processed files for CBF, ALFF, fALFF, ReHo, and DC. Given that the smoothed CBF data are at a resolution of 2 mm × 2 mm × 2 mm, they must be resampled to a resolution of 3 mm × 3 mm × 3 mm. This can be achieved by accessing the DPABI software package and selecting Utilities - Image Reslicer for resampling. Subsequently, the “Image Calculator” option should be chosen for further calculations. The correlation coefficients for CBF-ALFF, CBF-fALFF, CBF-ReHo, and CBF-DC are then calculated sequentially for each subject. A text file is generated for each indicator, and the values are ultimately extracted for statistical analysis. The multiple comparison correction is conducted using the False Discovery Rate (FDR) method.

### 2.6 Statistical Analysis

The sample size was calculated using Power Analysis and Sample size (PASS) software version 15, employing a two-tailed test with an alpha of 0.05 and a power of 0.95, maintaining an equal ratio of unilateral to bilateral participants (1:1). The primary observation indicators were CBF-ALFF, CBF-fALFF, CBF-ReHo, and CBF-DC, with calculations informed by data from our preliminary experiment.

The clinical data and mean values of NVC were analyzed using the Statistical Package for the Social Sciences, version 25.0. For the analysis of measurement data, independent sample t-tests were applied to data exhibiting a normal distribution, while the Mann-Whitney U-test was used for variables not following a normal distribution. Categorical data were analyzed using the Chi-squared test. Comparisons of mean NVC values between the two groups were conducted, with multiple comparisons adjusted using the FDR correction, maintaining a significance threshold of *P* < 0.05. A two-sample t-test was employed to evaluate differences in rs-fMRI parameters and CBF between the unilateral and bilateral asymptomatic MMD groups, utilizing a GLM. Subsequently, Spearman correlation analysis was performed to examine the associations between cognitive scores, diffusion values, and significant NVC alterations. An FDR correction was again applied to account for multiple comparisons, maintaining a significance level of *P* < 0.05.

## 3 RESULTS

### 3.1 Sample Size Calculation

The estimated sample size was at least 68, and our study included 119 participants, thereby adequately satisfying the accuracy requirements.

### 3.2 Demographic, Clinical Characteristics, and TBSS Results

Regarding gender, age, duration of education, vascular risk factors, and cognitive scores, no significant differences were observed between the unilateral and bilateral asymptomatic MMD groups, as indicated in Table **1**. However, when compared to the unilateral asymptomatic MMD group, the bilateral asymptomatic MMD group demonstrated a higher RD value (Fig. **2**, Table **2**) and a lower FA value (Fig. **3**, Table **3**) after family-wise error correction (*P* < 0.05). The specific brain regions showing these differences are detailed in the accompanying figures and tables.

### 3.3 3D pCASL, rs-fMRI, and NVC Results

The analysis revealed no statistically significant differences in CBF between the two groups, as determined by the FDR correction. In comparison to the unilateral asymptomatic MMD group, the bilateral asymptomatic MMD group exhibited both increased and decreased ALFF and fALFF in specific brain regions. Furthermore, ReHo and DC values were elevated in certain areas. Detailed information on these regional differences is provided in the supplementary figures and tables (Figs. **S1**-**S4**, Table **S1**-**S4**). Notably, the bilateral asymptomatic group exhibited significantly reduced global CBF-ALFF and CBF-ReHo coupling after FDR correction (*P* < 0.05) (Fig. **4**, Table **4**).

### 3.4 Correlation Analyses Results

Non-parametric Spearman correlation analysis revealed a positive correlation between CBF-ReHo coupling and FA in cluster 1 (*r* = 0.254, *P* = 0.005) (Fig. **5**), as well as between CBF-ALFF coupling and performance on the visual search task (*r* = 0.371, *P* = 0.003), and between FA in cluster 1 and performance on the verbal working memory-backward digit span task (*r* = 0.267, *P* = 0.031) (Fig. **6**). Additionally, there were negative correlations between CBF-ReHo coupling and RD in cluster 1 (*r* = -0.222, *P* = 0.025), and between CBF-ReHo coupling and RD in cluster 2 (*r* = -0.223, *P* = 0.018) (Fig. **5**). All results have been adjusted for the FDR to control for multiple comparisons.

## 4 DISCUSSION

 This study demonstrates that in asymptomatic MMD patients with normal signals, bilateral vascular involvement results in more severe NVC impairments, despite the absence of significant differences in CBF. This condition potentially causes cognitive decline by worsening white matter microstructural damage, thereby increasing the risk of adverse clinical outcomes as the disease advances. Consequently, the early detection of NVC dysfunction may serve as an indicator of heightened risk for future adverse clinical consequences and may inform early clinical decision-making regarding the necessity of vascular reconstruction surgery. These findings provide an imaging-based foundation for the selection of clinical strategies.

Under normal conditions, bilateral occlusion reduces CBF more significantly than unilateral occlusion [46-49], putting patients at higher risk of hypoperfusion. However, in some cases, collateral circulation or individual variability may preserve CBF in areas affected by bilateral occlusion [50, 51]. This study suggests that NVC dysfunction may occur before visible hemodynamic changes. Traditional CBF assessments mainly reflect perfusion after vascular structural changes [52-54], while NVC measures the functional response to neural activity of the neurovascular unit [55, 56]. Patients with bilateral vascular involvement showed lower NVC function than those with unilateral involvement, suggesting that the extent of vascular involvement directly affects the neurovascular compensatory reserve. This study shows that NVC sensitivity is higher than CBF in asymptomatic MMD patients.

Our findings reveal that asymptomatic patients with bilateral vascular involvement exhibit lower FA and higher RD values in certain brain regions compared with those with unilateral involvement, suggesting microscopic white-matter damage, including axonal and myelin disruptions. FA reflects the anisotropic diffusion of water molecules, making it crucial for assessing white matter integrity [57, 58]. A decrease in FA values is commonly interpreted as indicative of compromised axonal integrity [59]. RD is a significant marker for assessing myelin integrity [60]. An increase in RD is often regarded as indicative of myelin damage [61, 62], as it reflects enhanced diffusion of water molecules perpendicular to the axonal direction, typically associated with myelin degradation or injury [63, 64].

NVC reflects the close relationship between neural activity and CBF responses. This study found abnormalities in the rs-fMRI indicators that reflect spontaneous neuronal activity; however, there were no corresponding changes in CBF, providing evidence for a mismatch in NVC. Research has demonstrated that impairment of NVC function constitutes one of the initial pathological alterations in various diseases [65, 66]. A deficiency in NVC can precipitate cognitive decline when cerebral perfusion fails to meet the metabolic demands of neurons [67-69]. Empirical evidence suggests that under conditions of chronic hypoperfusion, the integrity of axon-glia interactions within white matter may be rapidly compromised, leading to a reduction in FA values and an increase in RD values [70]. These alterations may be linked to abnormalities in the microstructure of white matter, which subsequently impact cognitive function [71-73]. Meanwhile, the functionality of NVC is closely associated with the structural integrity of white matter and cognitive performance [74-77]. These findings align with our results, as our correlation analyses indicated that a reduction in NVC was significantly correlated with decreased FA values and increased RD values. Moreover, both the NVC dysfunction and the microstructural damage to white matter are partially linked to cognitive decline.

The findings of this study suggest that NVC serves as a crucial early indicator for evaluating cerebral blood flow regulatory reserve and stroke risk in asymptomatic individuals with MMD. The results indicate that individuals with bilateral vascular involvement are more prone to NVC functional uncoupling, reflecting a more significant impairment of their neurovascular compensatory mechanisms. From a pathophysiological standpoint, preserved NVC function implies that the cerebrovascular system remains capable of adequately responding to neuronal activity demands and maintaining sufficient oxygen supply, potentially delaying or preventing the onset of cerebral infarction. Conversely, NVC uncoupling indicates the depletion of cerebral blood flow regulatory capacity, thereby signifying an increased risk of stroke. Consequently, integrating macroscopic CBF assessment with NVC and DTI within a multimodal evaluation framework holds promise for facilitating earlier and more accurate identification of high-risk patients in future clinical practice, thus providing a critical opportunity for timely and individualized therapeutic interventions.

## 5 LIMITATION

This study is subject to several limitations. Firstly, it should be acknowledged that the investigation constitutes a preliminary exploration, constrained by a limited sample size. Second, the current set of cognitive measurements is not sufficiently comprehensive to cover the full range of cognitive abilities across all individuals. Thirdly, the study is constrained by the absence of longitudinal follow-up data. Future research endeavors should incorporate longitudinal observations to facilitate more thorough analyses. These limitations highlight the imperative for future large-scale, long-term studies that proactively collect longitudinal data, thereby enabling more in-depth analyses and providing robust, imaging-based evidence to inform clinical decision-making.

## CONCLUSION

 Investigating potential imaging changes in asymptomatic MMD is crucial before structural ischemic damage occurs. Even with normal brain signals, these patients show structural and functional impairments. NVC outperforms traditional CBF indicators in the early detection of brain dysfunction and may serve as a useful imaging biomarker for early cerebral injury in asymptomatic MMD.

## Figures and Tables

**Fig. (1):**
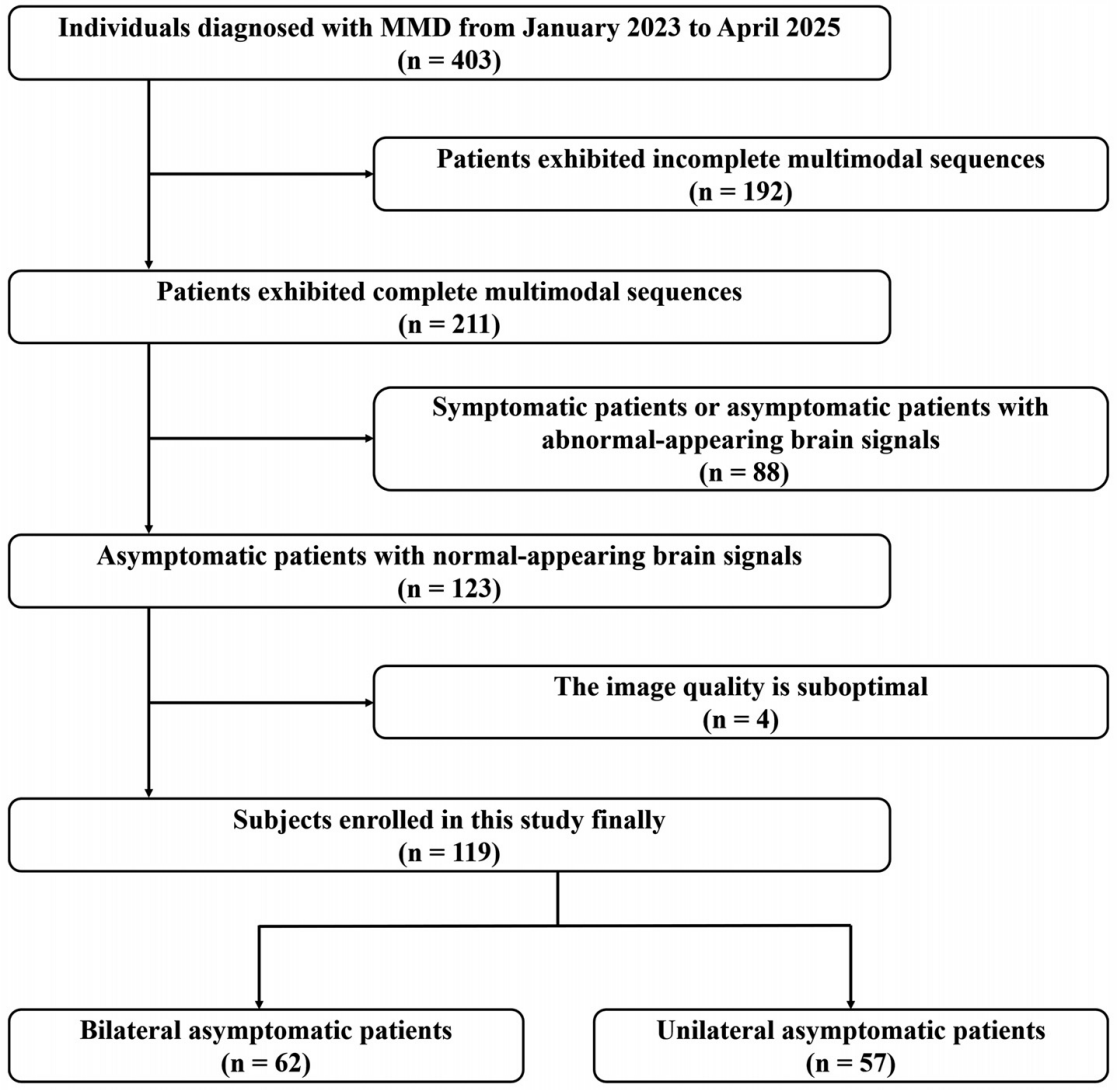
Flow diagram of participants in the study. MMD, moyamoya disease.

**Fig. (2):**
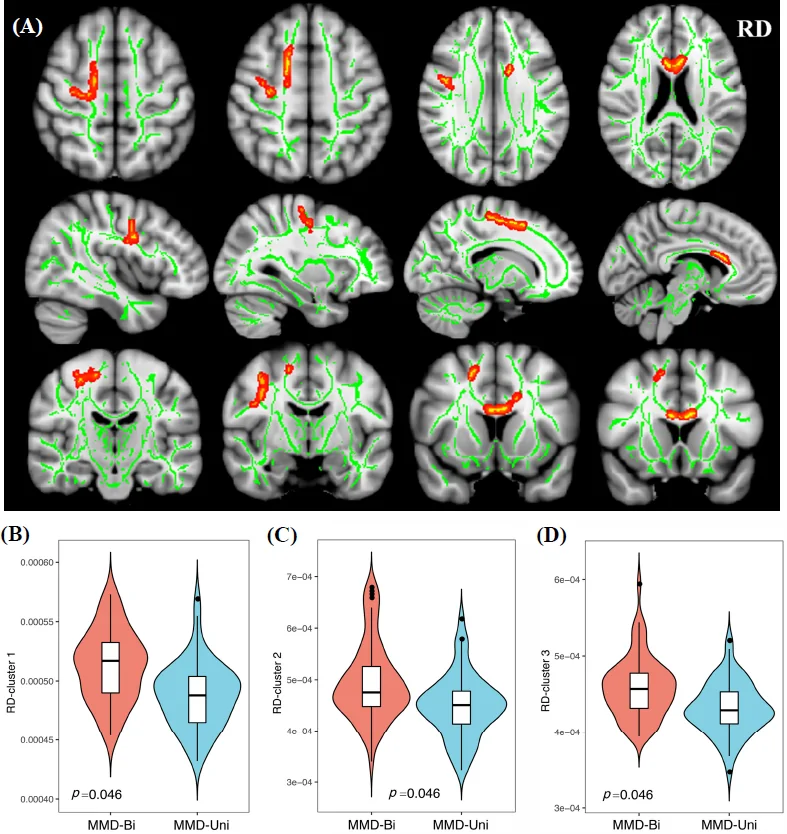
RD results of TBSS analyses. (**A**) Green indicates the average FA skeleton, while red indicates the regions where the mean RD value has increased in patients with MMD-Bi compared to those with MMD-Uni. (**B**-**D**) MMD-Bi showed a significantly increased mean RD value compared with MMD-Uni in three clusters, respectively. The *P* values have been adjusted for the family-wise error (*P* < 0.05). TBSS, tract-based spatial statistics; FA, fractional anisotropy; RD, radial diffusivity; MMD, moyamoya disease; Bi, bilateral vascular involvement; Uni, unilateral vascular involvement.

**Fig. (3):**
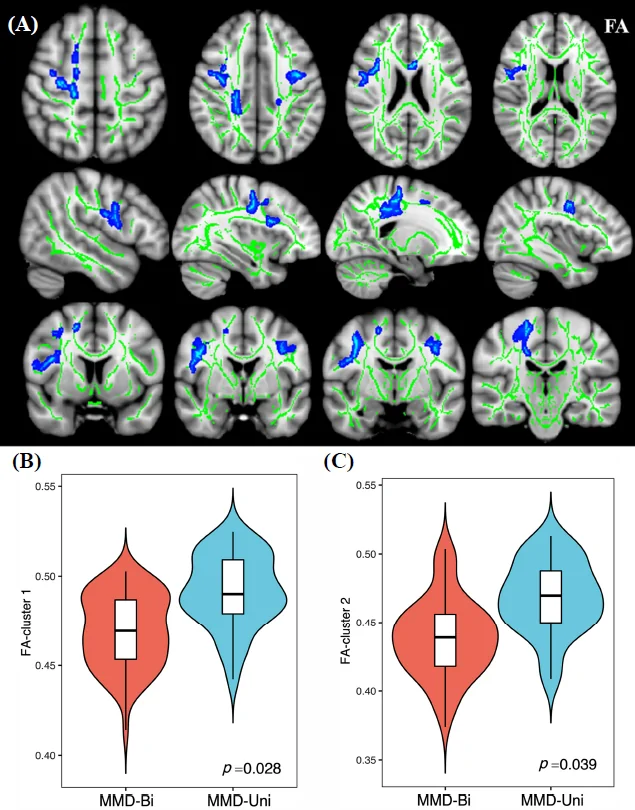
FA results of TBSS analyses. (**A**) Green indicates the average FA skeleton, while blue indicates the regions where the mean FA values has reduced in patients with MMD-Bi compared to those with MMD-Uni. (**B**, **C**) MMD-Bi showed a significantly decreased mean FA value compared with MMD-Uni in two clusters, respectively. The *P* values have been adjusted for the family-wise error (*P* < 0.05). TBSS, tract-based spatial statistics; FA, fractional anisotropy; MMD, moyamoya disease; Bi, bilateral vascular involvement; Uni, unilateral vascular involvement.

**Fig. (4):**
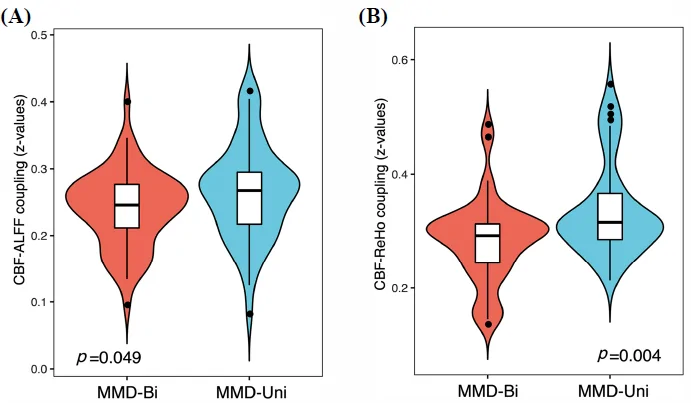
Results of NVC analyses. (**A**, **B**) MMD-Bi showed significantly decreased CBF-ALFF and CBF-ReHo coupling values compared with MMD-Uni. The *P* values have been adjusted for the false discovery rate (*P* < 0.05). NVC, neurovascular coupling; MMD, moyamoya disease; Bi, bilateral vascular involvement; Uni, unilateral vascular involvement. CBF, cerebral blood flow; ALFF, amplitude of low frequency fluctuation; ReHo, regional homogeneity.

**Fig. (5):**
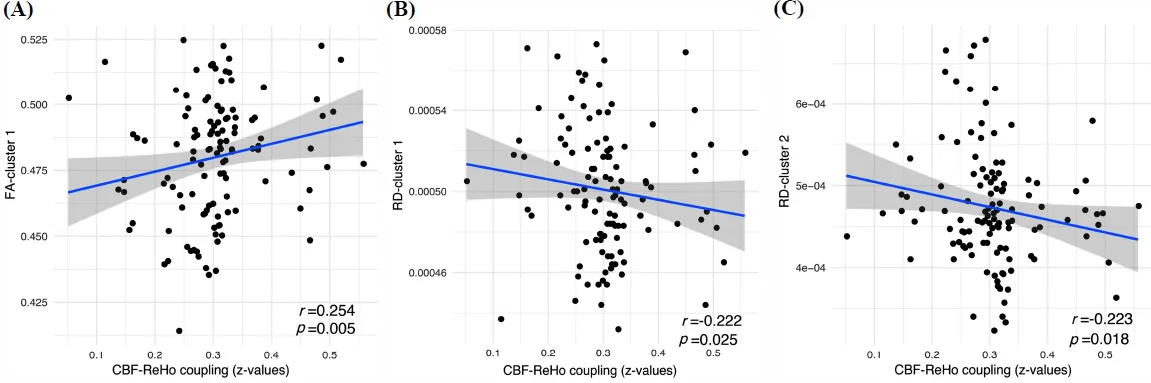
Correlation Analyses results. (**A**) CBF-ReHo coupling value was positively related to mean values of FA-cluster 1. (**B**, **C**) CBF-ReHo coupling value was negatively related to mean values of RD-cluster 1 and RD-cluster 2. The *P* values have been adjusted for the false discovery rate (*P* < 0.05). CBF, cerebral blood flow; FA, fractional anisotropy; RD, radial diffusivity; ReHo, regional homogeneity.

**Fig. (6):**
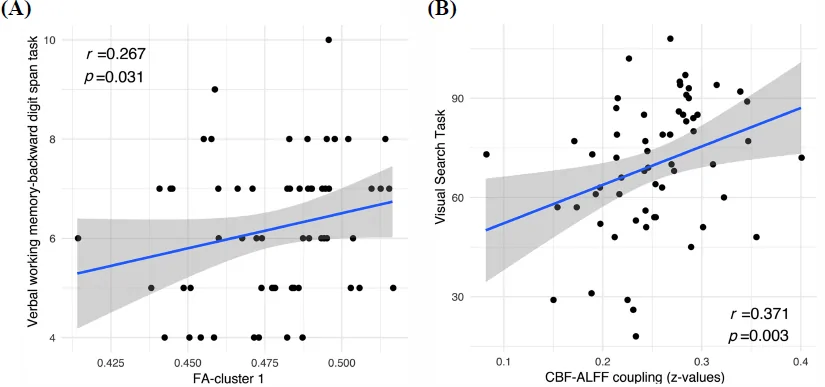
Correlation Analyses results. (**A**) The mean value of FA-cluster 1 was positively related to the verbal working memory-backward digit span task. (**B**) The CBF-ALFF coupling value was positively related to the visual search task. The *P* values have been adjusted for the false discovery rate (*P* < 0.05). FA, fractional anisotropy; ALFF, amplitude of low frequency fluctuation; CBF, cerebral blood flow.

**Table 1:** Participant demographic details, clinical information, and cognitive assessments.

**Variable**	**MMD-bilateral**	**MMD-unilateral**	**Statistical Value** (***x^2^*/*t*/*z***)	***P*-value**
Female sex, n (%)	29 (47)	35 (61)	2.557	0.110^c^
Age	38.95 ± 9.68	39.54 ± 10.06	-0.327	0.744^a^
Education years	12.00 (9.00,12.00)	12.00 (9.00,16.00)	-1.132	0.258^b^
Hypertension, n (%)	16 (26)	18 (32)	0.485	0.486^c^
Diabetes, n (%)	7 (11)	7 (12)	0.028	0.867^c^
Hyperlipidemia, n (%)	11 (18)	14 (25)	0.832	0.362^c^
Smoke behavior, n (%)	16 (26)	7 (12)	3.484	0.062^c^
Drinking behavior, n (%)	15 (24)	10 (18)	0.791	0.374^c^
CRT	397.62(370.00,487.00)	411.00(352.40,513.84)	-0.195	0.846^b^
VWM1	8.00 (7.00,9.00)	8.00 (7.75,9.00)	-0.106	0.916^b^
VWM2	6.00 (5.00,7.00)	6.00 (5.00,7.00)	-0.148	0.883^b^
Visual Search Task	67.45 ± 20.85	72.40 ± 18.83	-0.972	0.335^a^

**Note:** *^a^* Independent samples t-test (*t*). *^b^*Mann-Whitney U-test (*z*). *^c^*Chi-square test (*x^2^*). MMD: moyamoya disease, CRT: cognitive reflection test, VWM1: verbal working memory-forward digit span task, VWM2: verbal working memory-backward digit span task.

**Table 2:** Cluster sizes and locations for voxels with significantly increased RD values in bilateral MMD groups compared to unilateral MMD groups.

**Cluster/Voxel**	**JHU WM Tractography Atlas**	**Peak MNI Coordinate**			***t* Score**	***P* Value**
		**x**	**y**	**z**		
Cluster 1 / 522	Anterior thalamic radiation R	38	-7	33	2.921	0.046
-	Corticospinal tract R	-	-	-	-	-
-	Superior longitudinal fasciculus R	-	-	-	-	-
-	Superior longitudinal fasciculus (temporal part) R	-	-	-	-	-
Cluster 2 / 228	Cingulum (cingulate gyrus) L	0	10	22	3.554	0.046
-	Forceps minor	-	-	-	-	-
Cluster 3 / 168	Anterior thalamic radiation R	17	6	49	3.588	0.046
-	Superior longitudinal fasciculus R	-	-	-	-	-
-	Superior longitudinal fasciculus (temporal part) R	-	-	-	-	-

**Note:** RD, radial diffusivity; MMD, moyamoya disease; JHU WM, Johns Hopkins University white-matter; MNI, Montreal Neurological Institute; L, left; R, right. The *P* values have been adjusted for the family-wise error (*P* < 0.05).

**Table 3:** Cluster sizes and locations for voxels with significantly decreased FA values in bilateral MMD groups compared to unilateral MMD groups.

**Cluster/Voxel**	**JHU WM Tractography Atlas**	**Peak MNI Coordinate**			***t* Score**	***P* Value**
		**x**	**y**	**z**		
Cluster 1 / 1751	Anterior thalamic radiation L	40	-5	35	-3.709	0.028
-	Anterior thalamic radiation R	-	-	-	-	-
-	Corticospinal tract R	-	-	-	-	-
-	Cingulum (cingulate gyrus) R	-	-	-	-	-
-	Inferior fronto-occipital fasciculus R	-	-	-	-	-
-	Superior longitudinal fasciculus R	-	-	-	-	-
	Superior longitudinal fasciculus (temporal part) R		-	-	-	-
Cluster 2 / 356	Inferior longitudinal fasciculus L	-44	-4	36	-3.862	0.039
-	Superior longitudinal fasciculus L	-	-	-	-	-
-	Superior longitudinal fasciculus (temporal part) L	-	-	-	-	-

**Note:** FA, fractional anisotropy; MMD, moyamoya disease; JHU WM, Johns Hopkins University white matter; MNI, Montreal Neurological Institute; L, left; R, right. The *P* values have been adjusted for the family-wise error (*P* < 0.05).

**Table 4:** Between-group differences of global NVC.

**NVC**	**MMD-bilateral**	**MMD-unilateral**	**Statistical Value**(***t***)	***P*-value**	***P*-FDR Value**
CBF-ALFF	0.240±0.057	0.265±0.071	-2.111	0.037	**0.049** ^*^
CBF-fALFF	0.271±0.072	0.292±0.096	-1.369	0.174	0.696
CBF-ReHo	0.283±0.072	0.329±0.093	-2.960	0.004	**0.004** ^*^
CBF-DC	0.229±0.078	0.262±0.112	-1.822	0.072	0.144

**Note:** NVC, neurovascular coupling. MMD, moyamoya disease. The *P* values have been adjusted for the FDR (*P* < 0.05). FDR, false discovery rate. CBF, cerebral blood flow; ALFF, amplitude of low frequency fluctuation. fALFF, fractional amplitude of low frequency fluctuation. ReHo, regional homogeneity. DC, degree centrality.

## Data Availability

The datasets generated and analyzed during the current study are available from the corresponding author upon reasonable request.

## LIST OF ABBREVIATIONS

3D pCASL: Three-Dimensional Pseudocontinuous Arterial Spin Labeling
3D-T1: Three-Dimensional T1-Weighted Imaging
AD: Axial Diffusivity
ALFF: Amplitude of Low Frequency Fluctuation
CBF: Cerebral Blood Flow
CRT: Cognitive Reflection Test
DC: Degree Centrality
DTI: Diffusion Tensor Imaging
FA: Fractional Anisotropy
fALFF: Fractional Amplitude of Low Frequency Fluctuation
FDR: False Discovery Rate
FOV: Field of View
FSL: Software Library for Functional MRI
GLM: General Linear Model
MD: Mean Diffusivity
MMD: Moyamoya Disease
MNI: Montreal Neurological Institute
NVC: Neurovascular Coupling
PASS: Power Analysis and Sample Size
RD: Radial Diffusivity
ReHo: Regional Homogeneity
rs-fMRI: Resting-State Functional MRI
TBSS: Tract-Based Spatial Statistics
TE: Echo Time
TFCE: Threshold-Free Cluster Enhancement
TR: Repetition Time
VWM1: Verbal Working Memory–Forward Digit Span Task
VWM2: Verbal Working Memory–Backward Digit Span Task

## STANDARD OF REPORTING

STROBE guidelines were followed.
