# Supplementary material for: Neurovascular Coupling Dysfunction: An Early Indicator of Brain Injury in Asymptomatic Moyamoya Disease
Source: Curr Neuropharmacol. 2026 May 4;24(11):1777–89. doi: 10.2174/011570159X440563260226104506 (PMC13647728; doi:10.2174/011570159X440563260226104506)
Supplement: Supplementary file 1 [file CN-24-11-1777_SD1.pdf]

Supplementary Material

Neurovascular Coupling Dysfunction: An Early Indicator of Brain Injury in Asymptomatic Moyamoya Disease

Rui Hu[1,#], Jinhao Lyu[1,#], Ziqi Liu[2,#], Qi Duan[1], Chaobang Xie[1], Xiaokuan Hao[2], Caohui Duan[1], Song Wang[1], Xiangbing Bian[1], Jianxing Hu[1], Rong Wang[2], Lian Duan[3] and Xin Lou[1,*]

[1]Department of Radiology, The First Medical Center of Chinese PLA General Hospital, Beijing, China; [2]Department of Neurosurgery, Beijing Tiantan Hospital, Capital Medical University, Beijing, China; [3]Department of Neurosurgery, The Fifth Medical Center of Chinese PLA General Hospital, Beijing, China

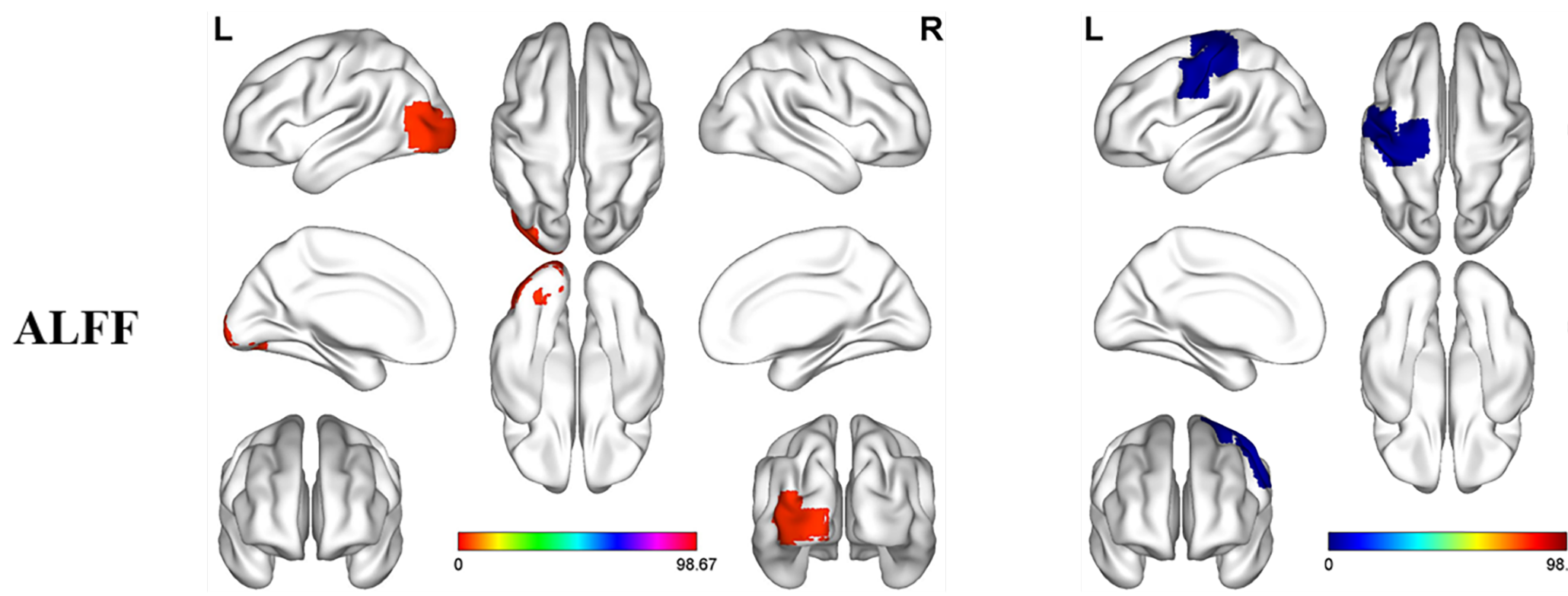

Figure S1. Comparisons of ALFF between unilateral and bilateral asymptomatic MMD show that regions with increased ALFF are depicted in red, whereas regions with decreased ALFF are depicted in blue. MMD, moyamoya disease; ALFF, amplitude of low frequency fluctuation.

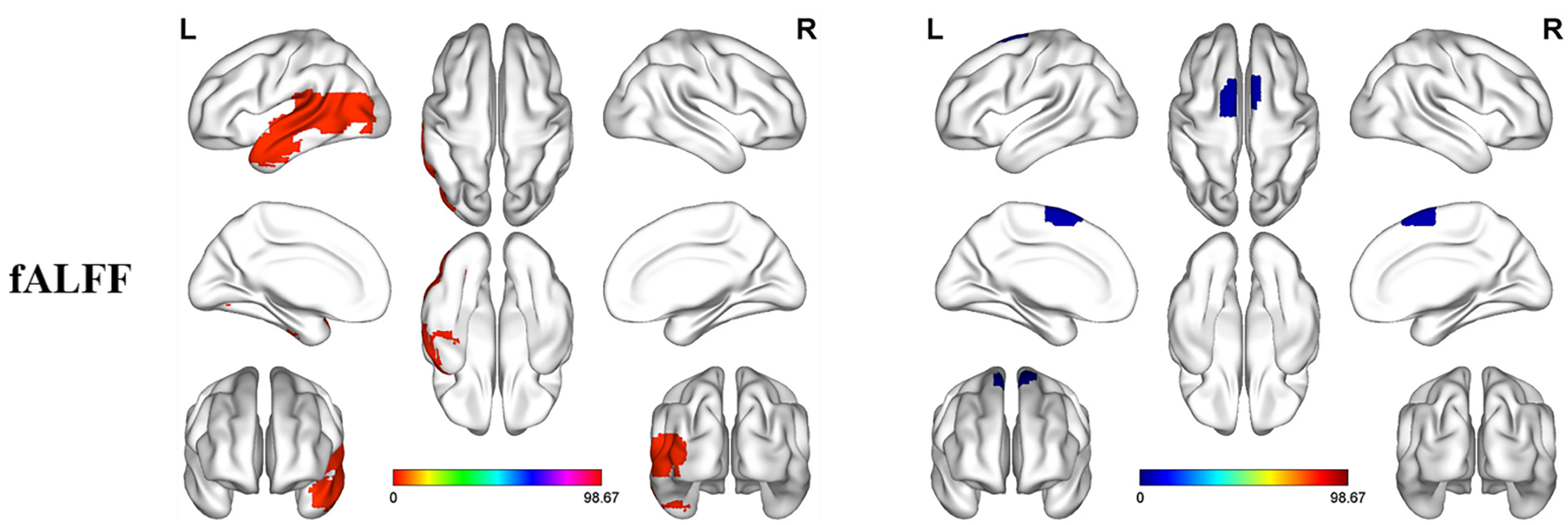

Figure S2. Comparisons of fALFF between unilateral and bilateral asymptomatic MMD show that regions with increased fALFF are depicted in red, whereas regions with decreased fALFF are depicted in blue. MMD, moyamoya disease; fALFF, fractional amplitude of low frequency fluctuation.

1875-6190/26 $65.00+.00 **© 2026 Bentham Science Publishers**

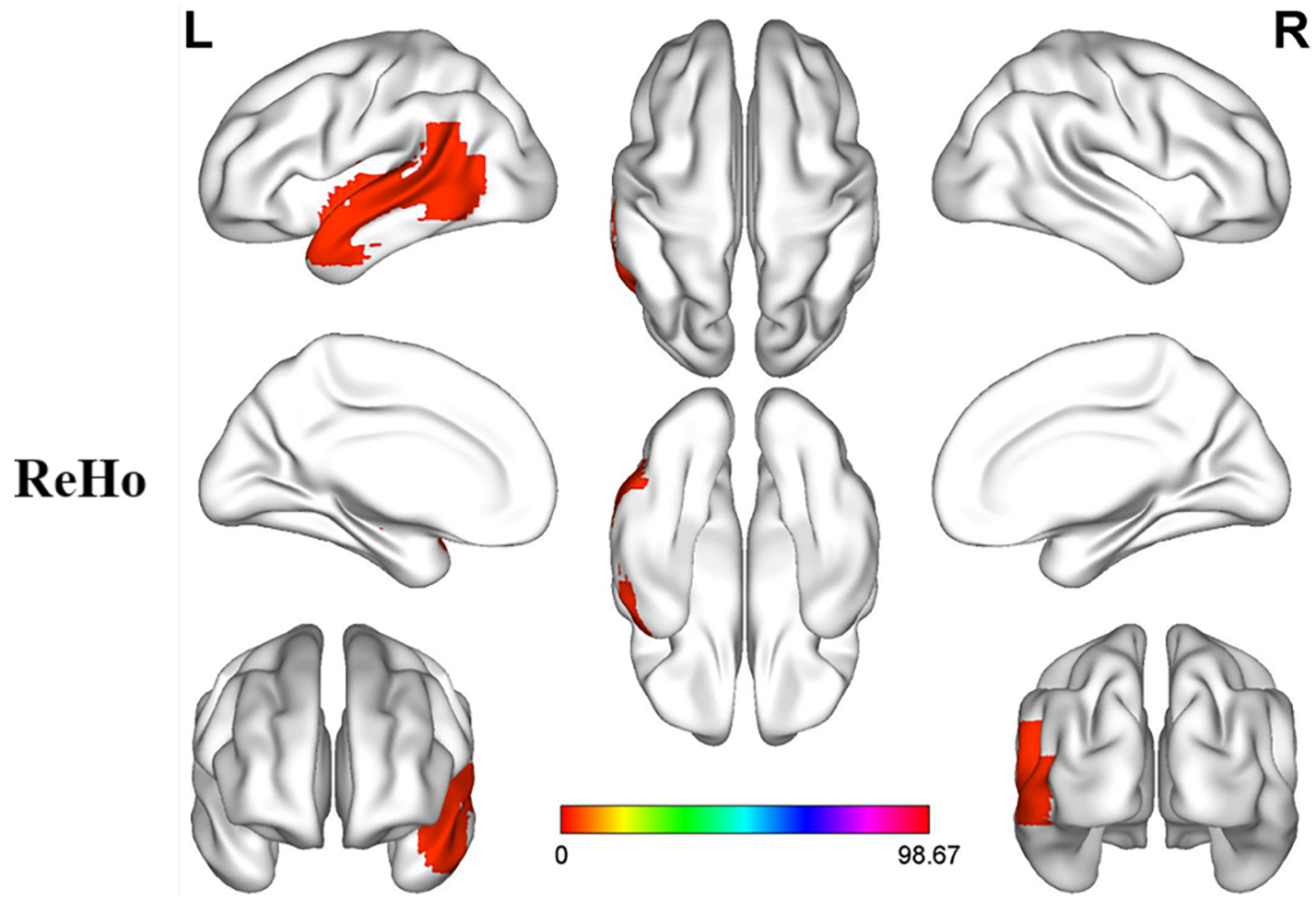

Figure S3. Comparisons of ReHo between unilateral and bilateral asymptomatic MMD show that red indicates regions with increased ReHo. MMD, moyamoya disease; ReHo, regional homogeneity.

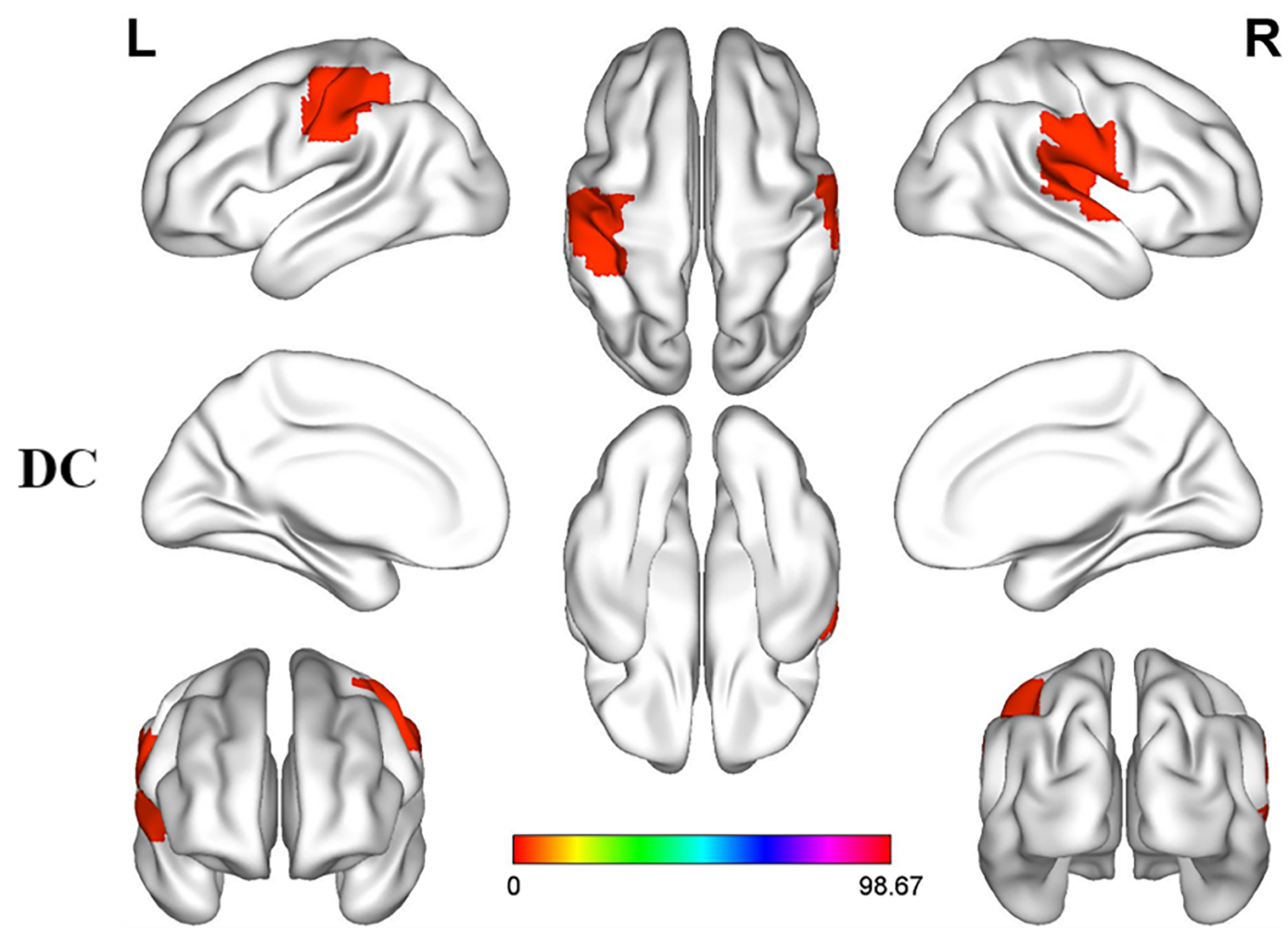

Figure S4. Comparisons of DC between unilateral and bilateral asymptomatic MMD show that red indicates regions with increased DC. MMD, moyamoya disease; DC, degree centrality.

Table S1. Brain regions with significant group differences in ALFF.

Cluster	Cluster Size	Peak MNI Coordinate			Brain Regions	Peak Intensity
		x	y	z		
ALFF (Bi>Uni)						
Cluster	115	-36	-78	-3	Occipital_Mid_L	4.9224
ALFF (Bi<Uni)						
Cluster	108	-57	-6	36	Postcentral_L	3.9394

MNI, Montreal Neurological Institute; Bi, bilateral vascular involvement; Uni, unilateral vascular involvement; ALFF, amplitude of low frequency fluctuation. Occipital_Mid_L, left Middle occipital gyrus; Postcentral_L, left Postcentral gyrus.

Table S2. Brain regions with significant group differences in fALFF.

Cluster	Cluster Size	Peak MNI Coordinate			Brain Regions	Peak Intensity
		x	y	z		
fALFF (Bi>Uni)						
Cluster	404	-48	-12	-9	Temporal_Mid_L	5.4224
fALFF (Bi<Uni)						
Cluster	59	-6	6	69	Supp_Motor_Area_L	4.9207

MNI, Montreal Neurological Institute; Bi, bilateral vascular involvement; Uni, unilateral vascular involvement; fALFF, fractional amplitude of low frequency fluctuation. Temporal_Mid_L, left Middle temporal gyrus; Supp_Motor_Area_L, left Supplementary motor area.

Table S3. Brain regions with significant group differences in ReHo.

Cluster	Cluster Size	Peak MNI Coordinate			Brain Region	Peak Intensity
		x	y	z		
ReHo (Bi>Uni)	305	-48	-3	-18	Temporal_Mid_L	5.647

MNI, Montreal Neurological Institute; Bi, bilateral vascular involvement; Uni, unilateral vascular involvement; ReHo, regional homogeneity. Temporal_Mid_L, left Middle temporal gyrus.

Table S4. Brain regions with significant group differences in DC.

Cluster	Cluster Size	Peak MNI Coordinate			Brain Regions	Peak Intensity
		x	y	z		
DC (Bi>Uni)						
Cluster 1	180	63	-12	27	Rolandic_Oper_R	4.5242
Cluster 2	121	-45	-30	45	Postcentral_L	4.0377

MNI, Montreal Neurological Institute; Bi, bilateral vascular involvement; Uni, unilateral vascular involvement; DC, degree centrality; Rolandic_Oper_R, right Rolandic operculum; Postcentral_L, left Postcentral gyrus.
